# Influence of systemic strontium ranelate on the progression and as adjunctive therapy for the nonsurgical treatment of experimental periodontitis

**DOI:** 10.4317/jced.58827

**Published:** 2021-12-01

**Authors:** David-Jonathan-Rodrigues Gusman, Henrique-Rinaldi Matheus, Breno-Edson-Sendão Alves, Edilson Ervolino, Nathália-Januario de Araujo, Bianca-Rafaeli Piovezan, Luiz-Guilherme Fiorin, Juliano-Milanezi de Almeida

**Affiliations:** 1 Department of Diagnosis and Surgery-Division of Periodontics, Sao Paulo State University (UNESP), School of Dentistry, Aracatuba, Sao Paulo, Brazil; 2 Nucleus of Study and Research in Periodontics and Implantology (NEPPI). São Paulo State University (Unesp), School of Dentistry, Araçatuba; 3 Department of periodontics, University of Western Sao Paulo (UNOESTE), Presidente Prudente, Sao Paulo, Brazil; 4 Department of Basic Sciences, School of Dentistry, Sao Paulo State University (UNESP), Aracatuba, Sao Paulo, Brazil

## Abstract

**Background:**

Strontium Ranelate (SR) presents overlapping osteoanabolic and anti-resorptive activity. However, the effects of SR on the progression of periodontitis through the alveolar bone and its potential applicability as adjunctive therapy to scaling and root planning remain poorly accessed. The aim of this study was to evaluate the effects of systemic (SR) both on the progression of experimental periodontitis (EP) and as adjunctive therapy to SRP.

**Material and Methods:**

Eighty male rats were divided into four groups (n=20): EP-PSS: EP induction and systemic administration of physiological saline solution (PSS); EP-SR: EP induction and systemic administration of SR; EP-SRP/PSS: EP induction, SRP and systemic administration of PSS; EP-SRP/SR: EP induction, SRP and systemic administration of SR. Seven days after ligature placement, SRP was performed in EP-SRP/PSS and EP-SRP/SR, as well as the systemic administration of either PSS or SR were initiated and continued until euthanasia in all groups. Animals were euthanized at 7 and 30 days after the beginning of the systemic treatments. Histological, histometric (percentage of bone in the furcation [PBF]) and immunohistochemical (tartrate-resistant acid phosphatase [TRAP], Osteocalcin [OCN] and leukocyte common antigen [CD 45]) analyses were performed. Data were statistically analyzed.

**Results:**

EP-SRP/PSS showed a significantly more organized pattern of the connective tissue and alveolar bone structure than EP-SRP/SR. EP-SR showed significantly higher PBF than EP-PSS, however, EP-SRP/PSS showed no difference with EP-SRP/SR at 30 days.

**Conclusions:**

SR reduced the alveolar bone loss in non-treated animals and presented no standout benefits over the conventional forms of treating EP.

** Key words:**Strontium Ranelate, periodontal disease, root planing, alveolar bone loss.

## Introduction

The prevalence of periodontitis ranges from 20 to 50% of the worldwide population and has been reported as the most prevalent bone-loss related disease associated in adults (1). The gold standard for the treatment of periodontitis relies on the removal of pathogenic subgingival microbiota through scaling and root planning (SRP) (2). However, systemic modifying factors, such as diabetes mellitus, tobacco smoking, and immunosuppression affect its pathogenesis and is likely to reduce the gains expected to be achieved with SRP (3). Also, compromising local factors (e.g. root depressions, root concavities, furcation areas, and cervical enamel projections) jeopardize periodontal repair following SRP (4). A challenging scenario that encompasses one or more of these conditions triggers the necessity of adjunctive therapies to SRP (5).

Different substances have been proposed as adjunctives to SRP. Anti-resorptive drugs presented positive effects both in progression (i.e. no treatment conducted) of experimental periodontitis and as adjunctive to SRP (6). Strontium (Sr) is an alkaline earth metal, atomic number 38, belonging to the second group of the periodic Table (7). The physicochemical similarities between calcium and Sr enable this compound to be incorporated into the mineral phase of bone (8), mainly deposited during ossification or at remodeling trabecular seams (9). Furthermore, Sr develops important roles in fundamental processes related to bone metabolism by promoting osteoblast-mediated bone formation and inhibition of osteoclast-mediated bone resorption (10).

Strontium Ranelate (SR) has been used for treatment of postmenopausal osteoporosis (11). The molecule of SR is composed of two stable atoms of Sr (Sr+2), and its inactive segment corresponds to a synthetic organic molecule chemically presented as 5-(bis[carboxymethyl] amino) -2-carboxy-4-cyano-3-thiophenacetic (12). Although the mechanisms of action of SR to remain not totally elucidated, *in vitro* studies showed that SR increases the osteogenic differentiation of bone marrow stromal cells, stimulates osteoblastic differentiation of human mesenchymal stem cells, increases osteoblast proliferation/differentiation, reduces osteoblast apoptosis, decreases osteoclast differentiation/activation and increases apoptosis of osteoclasts (13). Also, *in vivo* assessments reported positive effects of SR over the skeleton, since it was related with increased bone mass density, improvement on the mechanical properties and bone strength, and reduction of new vertebral and nonvertebral fractures (13).

Currently, few evidence have been published regarding the effects of SR intake over the alveolar bone loss in non-systemically modified animals induced to experimental periodontitis (EP)(6,14). Souza *et al*. (14) concluded with a rodent model of EP induction with nylon threads in maxillary first molars that both 20 and 100 mg/kg of SR prevented alveolar bone loss. On the other hand, the results of Karakan *et al*. (6) indicate that the benefits of SR seem to be dose-dependent, since the prevention of alveolar bone loss in lower mandibular first molars induced to EP with silk threads was achieved with a dose of 900 mg/kg of SR (doses tested were 300, 625 and 900 mg/kg). Additionally, Karakan *et al*. (6) hypothesized that SR could be used as an adjunctive to mechanical therapy.

The beneficial properties of SR over the fundamental processes of bone formation and bone resorption may consist of important features during the periodontal repair following SRP, and, to the best of our knowledge, no studies have accessed this topic. Therefore, the aim of this study was to evaluate the effects of systemic SR on the progression of EP, as well as, adjunctive therapy to SRP.

## Material and Methods

-Animals

This study followed a randomized, single-blind, controlled design, and was conducted in accordance with the ARRIVE Guidelines: Animal Research: Reporting *In Vivo* Experiments (15). The experimental protocol was approved by the Ethics Committee on Animal Use under protocol 2018-00590 of São Paulo State University (UNESP, School of Dentistry, Araçatuba). Eighty healthy 3-month-old male rats (Rattus norvegicus, albinus; Wistar), weighing 250–300 g, were used. They were kept in plastic boxes with wood shavings, under 12 hr/12 hr light/dark cycles, 22 ± 2°C ambient temperature, 20 air changes per hour, 55 ± 5% humidity, receiving feed and water *ad libitum*, and monitored daily.

-Sample size, randomization, and experimental groups

Sample size was calculated based on previous literature (16) to achieve a 0.8 power and 0.05 alpha error based on a 12% potential standard deviation and the assumption that a 10% difference between groups/periods would be relevant. To compensate possible dropouts, the sample size was estimated to be n = 10.

Numbers from 1 to 80 were labeled in the upper tail of the animals, the number sequence was uploaded to the Minitab® 17 software (Minitab Inc., State College, PA, USA), and simple randomization (1:1 allocation ratio) was performed using a computer-generated random number Table by a masked staff not involved with the study. The animals were assigned to one of the four experimental groups:

• Group EP-PSS (n = 20) – EP induction and systemic administration of physiological saline solution (PSS);

• Group EP-SR (n = 20) – EP induction and systemic administration of SR;

• Group EP-SRP/PSS (n = 20) – EP induction, SRP and systemic administration of PSS;

• Group EP-SRP/SR (n = 20) – EP induction, SRP and systemic administration of SR.

-Anesthesia

The surgical interventions were stated by sedation and general anesthesia, obtained by via intra-muscular injection with xylazine hydrochloride (Vetaset; Zoetis, Florham Park, NJ, USA) (6 mg/kg of body weight) and ketamine hydrochloride (Coopazine; Coopers, São Paulo, SP, Brazil) (70 mg/kg of body weight).

-Experimental periodontitis induction

At day 0, the experimental periodontitis was induced by the placement of a #24 cotton thread (Cotton Chain No. 24; Coats Corrente, São Paulo, SP, Brazil) around the mandibular left first molars of all animals (16).

-Scaling and root planning 

At day 7, the #24 cotton thread was removed from groups EP-SRP/PSS and EP-SRP/SR. Then, SRP was performed with manual curettes (#1-2 Mini Five Gracey curettes, Hu-Friedy, Chicago, IL, USA) through 10 distal-mesial traction movements in both the buccal and lingual aspects. The furcation and interproximal regions of each tooth were scaled with the same curettes through cervical-occlusal traction movements (5).

-Systemic treatment 

SR was dissolved in mineral water (Crystal, Anápolis, GO, Brazil) at the dose of 900 mg/kg of SR (Protos®, Servier, Gidy, France) and the suspension was administered by gastric gavage, as soon as prepared. In the placebo group, 0.3 ml of PSS (Equiplex Indústria Farmacêutica, Aparecida de Goiânia, GO, Brazil) was administered by gastric gavage.

The administration of either SR and PSS was initiated at day 7 and were continued daily until the euthanasia. A 38 mm length curved stainless-steel needle attached to a syringe (volume: 1 mL) was used to inject the solutions directly into the gastrointestinal tract. Animals were weighed daily on an electronic scale in order to SR be administered at the proper dosage (17).

-Euthanasia

The euthanasia were performed at 7 and 30 days after initiating the systemic administration of either SR or PSS, with an overdose (150 mg/kg) of sodium thiopental (Cristália Ltda., Itapira, SP, Brazil).

-Tissue processing

The left hemimandibles were fixed in buffered 4% formaldehyde for 48 hr, demineralized buffered 10% ethylenediaminetetraacetic acid (EDTA) solution, and subsequently submitted to histological processing and paraffin embedding. Five semi-serial 4 μm thickness obtained from the central furcation region in a mesial-distal direction were stained with haematoxylin and eosin (H&E) for histologic and histometric analyses. Six additional sections from each specimen were subjected to the indirect immunoperoxidase method, using the following primary anti-bodies: goat anti-tartrate-resistant acid phosphatase (TRAP) (1:100; SC1252; Santa Cruz Biotechnology, Santa Cruz, CA, USA), goat anti-osteocalcin (OCN) (1:200; SC1350; Santa Cruz Biotechnology, Santa Cruz, CA, USA) and goat anti-leukocyte common antigen (CD 45) (1:100, SC 7628; Santa Cruz Biotechnology, Santa Cruz, CA, USA). As negative control, the sections underwent the same procedures suppressing the primary antibodies. Immunohistochemical processing was conducted according to Gusman *et al*. (16).

-Analysis of the results

All analyzes were performed by examiners who were calibrated and masked with respect to the experimental groups and periods, by using image analysis software (AxioVision 4.8.2; Carl Zeiss MicroImaging GmbH, Jena, TH, Germany).

-Histopathological analysis

The histopathological analysis of the periodontal tissues encompassed the mesial, the furcation and the distal regions of the lower left first molars. It was conducted by a certified histologist (EE) in accordance with an adaptation of the criteria established by Gusman *et al*. (16) The parameters of (a) intensity of local inflammatory response; (b) extension of inflammatory infiltrate; (c) pattern of the connective tissue structure; and (d) pattern of the alveolar bone structure were evaluated and each specimen was assigned to the scores 0 to 3 within each parameter, as shown in Table 1.

**Table 1:**
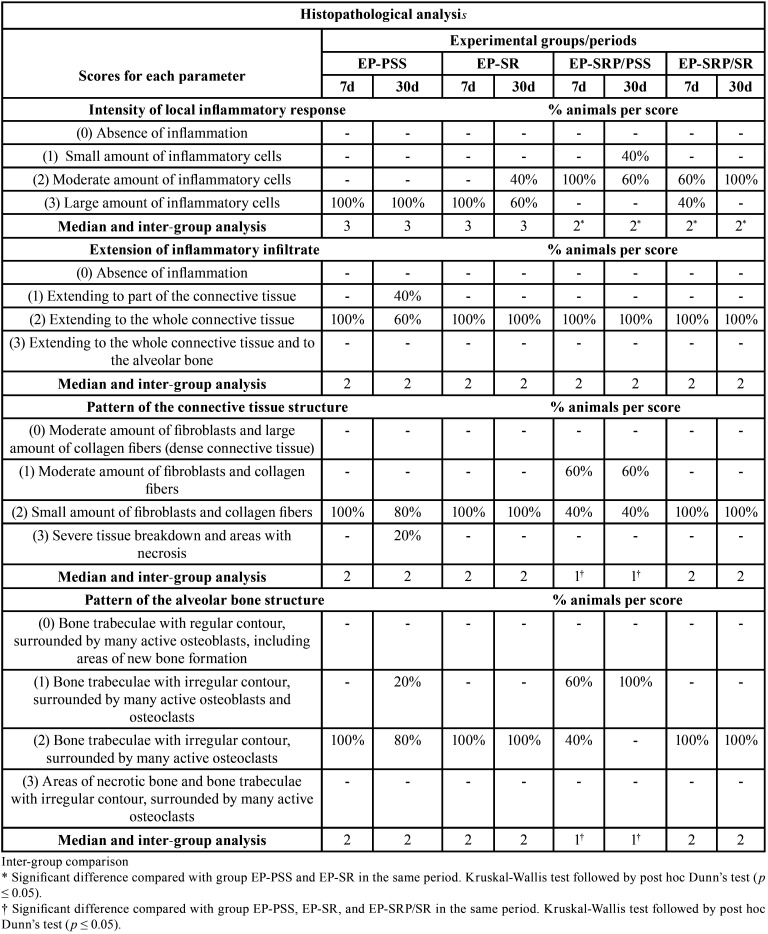
Scores and specimens’ distribution according to the parameters of the histopathological analysis in the left mandibular first molar in each group and period.

-Histometric analysis

For the histometric analysis, the entire furcation area (FA) was delineated, measured in mm2, and considered 100% of the area to be analyzed. Then, the bone area was measured (mm2) within the limits of FA. The ratio of BA to FA was calculated and expressed as the percentage of bone in the furcation (PBF)(16). Intra-examiner reliability and reproducibility was carried out with remeasurement of the 4 sections stained with HE 1 week after the first measurement.

-Immunohistochemical analysis

The immunohistochemical analysis was performed under light microscopy at × 400 magnification. The number of TRAP-positive cells with three or more nuclei was counted in the central area of the inter-radicular septum within an area of 1600 x 1200 µm. The coronary limit was the bone crest, which was spanned apically for a distance of 1200 µm.

A semi-quatitative analysis of the immunolabeling of BMP2/4 and OCN was carried within the FA. Criteria based on the work of Gusman *et al*. (16) were characterized as follows:

• Score 0: no immunolabelling (total absence of immunoreactive [IR] cells);

• Score 1: low immunolabelling (IR in ~1/4 of cells per area);

• Score 2: moderate immunolabelling (IR in ~1/2 of cells per area);

• Score 3: high immunolabelling (IR in ~3/4 of cells per area).

-Statistical analysis

Data were analyzed using BioSstat software (BioStat version 5.0, Belém, PA, Brazil). For the parameters assessed in the histological and immunohistochemical analyses of OCN and CD45, the significance of the differences among groups was determined by a Kruskal–Wallis test, followed by a post hoc Dunn’s test (*p* ≤ 0.05). The normality and homogeneity of variances were verified for PBF and TRAP parameters using the Shapiro–Wilk test. The significance of differences among groups was determined by one-way analysis of variance, followed by a post hoc Tukey’s test (*p* ≤ 0.05).

## Results

-Histological and histometric analyses

**Figure 1:**
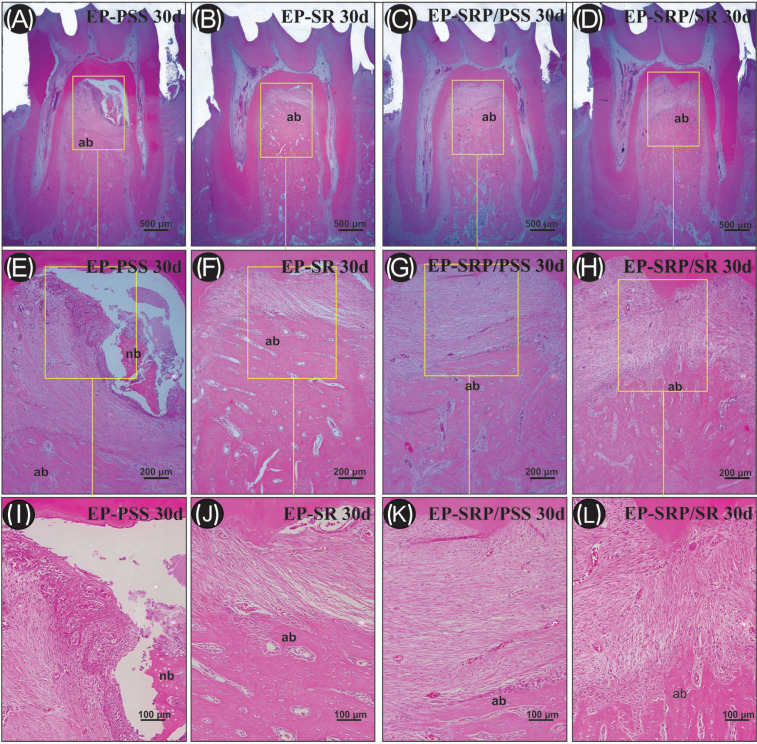
Photomicrographs of the furcation region of the mandibular left first molar of all groups at 30 days. All groups showed alveolar bone loss and inflammatory infiltrate and EP-PSS showed the highest alveolar bone loss. Abbreviations: ab: alveolar bone; d: days; EP, experimental periodontitis; nb: necrotic bone; PSS, physiological saline solution; SR: Strontium Ranelate; SRP: scaling and root planning; μm, micrometers. Haematoxylin and eosin staining. Original magnification: A-D, ×25; E-H, x100; I-L, 200x. Scale bars: A-D, 500 μm; E-H, 200 μm; I-L,100 μm.

Regarding the histological analysis, EP-PSS and EP-SR showed significantly more inflammatory cells (p≤ 0.05) than EP-SRP/PSS and EP-SRP/SR at 7 and 30 days (Figure 1A through 1L). Despite distinct intensities among groups, the inflammatory infiltrate was always extended through the whole connective tissue (Fig. 1A through 1L) (Table 1).

With regard to the structural pattern of the connective tissue and alveolar bone, EP-SRP/PSS showed connective tissue arranged with moderate amount of fibroblast and collagen fibres, and bone trabeculae with irregular contour, surrounded by many active osteoblasts and osteoclasts at 7 and 30 days (Figure 1C, G, and K), while in EP-PSS, EP-SR and EP-SRP/SR was observed small amount of fibroblasts and collagen fibers and bone trabeculae with irregular contour, surrounded by many active osteoclasts (Fig. 1A, B, D, E, F, H, I, J, L) (Table 1).

**Figure 2:**
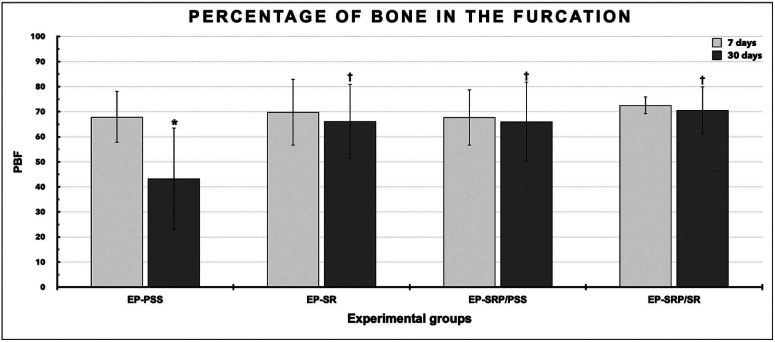
Means and standard deviations (M ± SD) of the percentage of bone in the furcation region of the left mandibular first molar in all groups 7 and 30 days. Abbreviations: EP, experimental periodontitis; PSS, physiological saline solution; PBF, percentage of bone in the furcation; SRP, scaling and root planning; SR, Strontium Ranelate. Symbols: *, Significant difference compared with 7 days in the same group. †, Significant difference compared with group EP-PSS in the same period. Statistical test: ANOVA followed by post-hoc Tukey’s test (*p* ≤ 0.05).

The Kappa test indicated a high level of agreement (96.2%) in the intra-examiner measurements of PBF. Means and standard deviations of the PBF are presented in Figure 2. The intragroup comparisons showed that PBF was significantly higher in group EP-PSS at 7 days when compared with 30 days. For the intergroup comparisons, the values of PBF in groups EP-SR, EP-SRP/PSS and EP-SRP/SR were significantly higher when compared with group EP-PSS at 30 days.

-Immunohistochemical analyses

**Figure 3:**
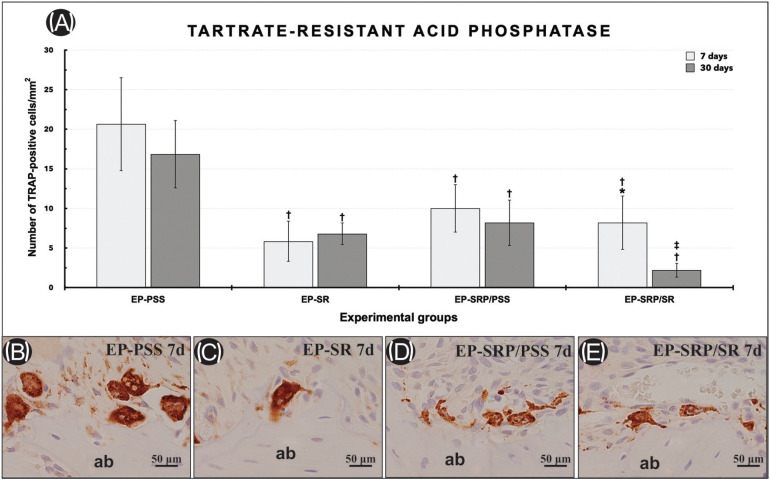
Means and standard deviations (M ± SD) of the TRAP-positive cells (cell/mm2) in the furcation area of the left mandibular first molar in all groups at 7 and 30 days (A). Symbols: *, Significant difference compared with 30 days in the same group; †, Significant difference compared with group EP-PSS in the same period; ‡ Significant difference compared with group EP-SRP/PSS in the same period; Statistical test: ANOVA followed by post-hoc Tukey’s test (*p* ≤ 0.05). Photomicrographs showing immunolabelling of TRAP-positive cells in the furcation area of the left mandibular first molar in all groups at 7 days (B-E). Abbreviations: ab: alveolar bone; d: days; EP, experimental periodontitis; PSS, physiological saline solution; SR: Strontium Ranelate; SRP: scaling and root planning; μm, micrometers. Original magnification: ×400. Counterstaining: Harry’s haematoxylin. Scale bars: B-E, 50 μm.

**Figure 4:**
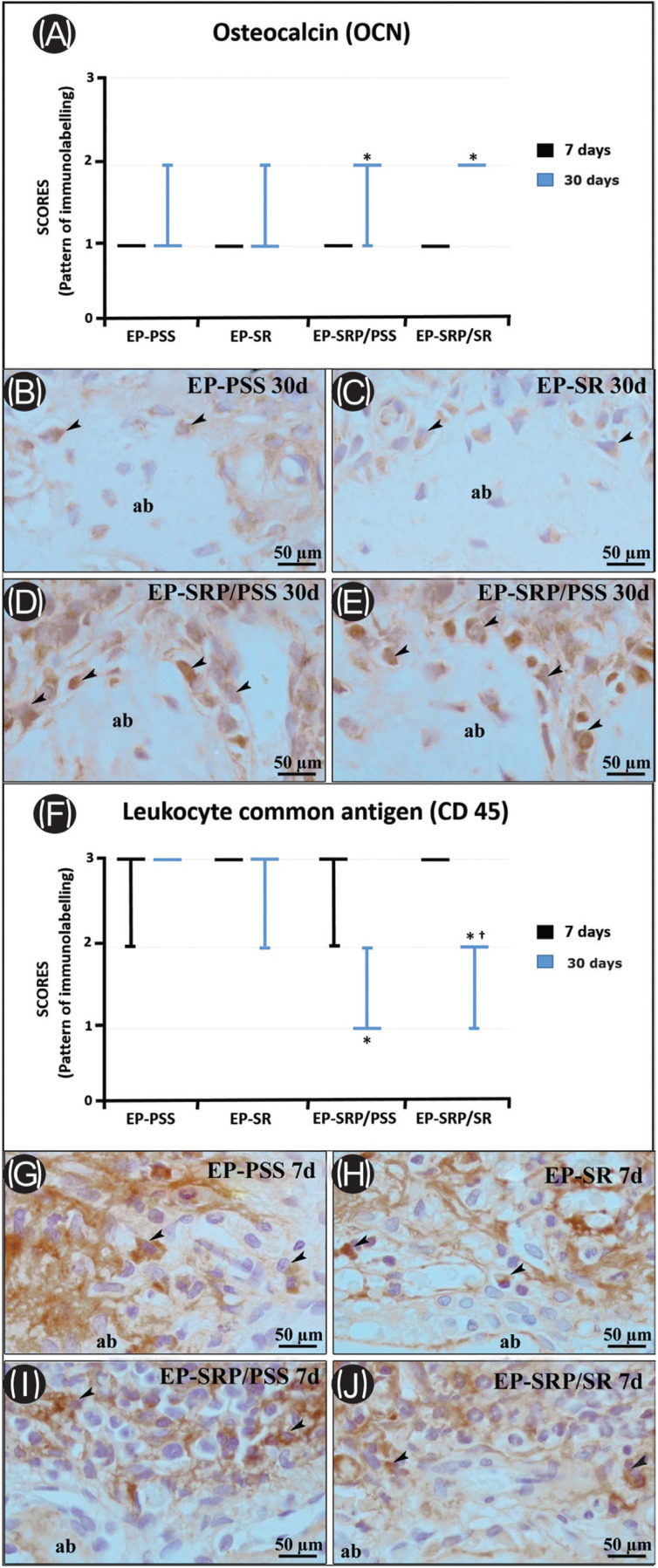
Graphics showing the median and interquartile range of the scores for OCN (A), CD45 (F) in the furcation region of the mandibular left first molar, assigned to each group and period. Symbols: *, significant difference compared with EP-PSS and EP-SR in the same period; †, significant difference compared with EP-SRP/PSS in the same period. Kruskal–Wallis test followed by post hoc Dunn’s test (p ≤ 0.05). Photomicrographs showing immunolabelling pattern of OCN (B-E) in the furcation area of the left mandibular first molar in all groups at 30 days. Photomicrographs showing immunolabelling pattern of CD-45 (G-J) in the furcation area of the left mandibular first molar in all groups at 7 days. Abbreviations: ab: alveolar bone; d: days; EP, experimental periodontitis; PSS, physiological saline solution; SR: Strontium Ranelate; SRP: scaling and root planning; μm, micrometers. Black arrows, immunolabelling cells. Original magnification: ×400. Counterstaining: Harry’s haematoxylin. Scale bars: 50 μm.

The immunohistochemical techniques used for detecting TRAP, OCN, and CD45 yielded high specificity in the detection of these proteins, as evidenced by the total absence of immunolabelling in the negative controls. The immunolabelled cells had a brownish color that was confined to the cytosolic compartment in TRAP and confined to the cytosolic compartment and poorly to the extracellular matrix in OCN and CD45. All groups presented immunolabels for TRAP (Fig. 3-E), OCN (Fig. 4B-D, and E) and CD45 at all periods of analysis (Fig. 4G- J).

Means and standard deviations of the number of TRAP (cell/mm2) at 7 and 30 days, with inter-group and intra-group comparisons, are presented in Figure 3A. In the intra-group comparisons, EP-SRP/SR showed higher number of TRAP-positive cells at 7 days when compared with 30 days. In the inter-group comparison, EP-SR, EP-SRP/PSS and EP-SRP/SR showed higher number of TRAP-positive cells than EP-PSS at 7 and 30 days (Fig. 3B through E, at 7 days). EP-SRP/PSS showed higher number of TRAP-positive cells when compared with EP-SRP/SR at 30 days.

Scores, medians, and specimen’s distribution for OCN and CD45 immunolabelling at 7 and 30 days, with inter-group comparisons, are presented in Figure 4A (OCN) and Figure 4F (CD45). EP-SRP/PSS and EP-SRP/SR showed significantly higher OCN immunolabeling than EP-PSS and EP-SR at 30 days (Fig. 4B throughout E). EP-SRP/PSS and EP-SRP/SR showed significantly lower CD45 immunolabeling than EP-PSS and EP-SR at 30 days. Furthermore, EP-SRP/SR showed significantly higher CD45 immunolabeling than EP-SRP/PSS at 30 days. No intergroup significant difference was observed at 7 days with regard to OCN and CD45 immunolabeling (Fig. 4G throughout J).

## Discussion

Animal experimentation is necessary for *in vivo* validation, since *in vitro* testing may not be sufficient to access the complexity of some biological events and human studies present results which mechanisms are difficult to prove (18). Different methods have been proposed to access EP (5,18-20), mainly in rodent models, due to its easy management, low cost, and the similarity between rats’ and humans’ biological response (21,22). Moreover, this model of EP induction in rats’ mandibular molars mimics challenging conditions of the clinical scenario, since the reduced dimensions of these teeth represent areas of difficult access for mechanical instruments. Therefore, this model may be considered appropriate for the evaluation of adjuvant therapies (5). The experimental model of this research was used to investigate both situations, progression and treatment of EP, and was carried out under the principles of reduction, refinement and replacement for the laboratory use of animals.

Two previous researches investigated the effects of SR on the EP in healthy rats, however, by using distinct models (6,14). Karakan *et al*. (6) followed a design of subgingival placement of silk threads around the mandibular first molars, which is more usual in the literature. On the other hand, Souza *et al*. (14) used a nylon thread around maxillary molars in order to induce EP. These experiments also differed with regard to the doses of SR administered to the animals. Karakan *et al*. (6) showed the best results with the highest tested dose (900 mg/kg), while Souza *et al*. (14) showed 38% prevention of bone loss by using 100 mg/kg.

The exact mechanisms of action of the SR remain unclear. Evidence indicates that SR act by inducing osteoblastic differentiation via stimulation of calcium sensor receptor inhibiting the osteoclastic differentiation by RANKL suppression, and promoting of osteoprotegerin (OPG) activity (6).

With regard to the progression of EP under the influence of the systemic administration of SR, although no significant differences were observed in the histopathological analysis, the histometric analysis showed a higher PBF in group EP-SR when compared with EP-PSS at 30 days. The presence of higher amount of bone in EP-SR may not be due its formation, but to decreased bone resorption, since EP-SR expressed lower number of TRAP-positive cells than EP-PSS at 7 and 30 days. The feature of SR preventing alveolar bone loss in EP had already been accessed by Karakan *et al*. (6) who also observed reduced number of osteoclasts in the tested groups. Therefore, based on the interpretation of the results of the histopathological and immunohistochemical analyzes, it can be inferred that the paths by which EP disrupts periodontal apparatus is similar in EP-SR and EP-PSS, however with lower bone loss in EP-SR due to the decreased expression of TRAP-positive cells at 7 and 30 days.

The anti-inflammatory property of SR has been described in an osteoarthritis (OA) environment, where it displays an indirect role in the cartilage by delaying OA progression via downregulation of MMP-2 and MMP-9 (23,24). In dentistry, a rodent model of zymosan-induced temporomandibular joint inflammation observed that SR may present antinociceptive and inti-inflammatory effects by reducing TNF-α levels in the trigeminal ganglion (25).

Heme-Oxygenase (HO)-1 plays an important downregulatory role in cellular activation and expression of inflammatory mediators, thus, modulating the inflammatory process (26) Souza *et al*. (30) showed that SR elevated the expression of HO-1 in EP, inferring an anti-inflammatory property of this drug in the periodontium. On the other hand, the histopathological parameters and the evaluation of the CD45 showed no significant differences between groups EP-SR and EP-PSS at 7 days. The results of the present research corroborated with Karakan *et al*. (6) who reported that no difference was observed in serum IL-1β between control group and SR group.

The lack of consensus on the anti-inflammatory property of SR may be substantiated by the distinctive metabolism among the analyzed tissues and by the complexity of the mechanisms enrolled with the pathogenesis of periodontitis (27). The periodontal collapse is the consequence of plaque accumulation, release of bacterial substance, and hosts’ response, which lead to deeper and progressive pocket depths and more challenging scenarios for treatment (28).

The semi-quantitative evaluation assessing if systemic SR could either improve or not the periodontal repair following SRP showed a significantly more structured pattern of the connective tissue and alveolar bone in EP-SRP/PSS when compared with EP-SRP/SR in all periods of analysis, with no difference in the PBF between both groups. Also, animals from group EP-SRP/PSS expressed lower immunolabelling pattern of CD45 than EP-SRP/SR at 30 days.

The restoration of tissues’ architecture is dependent of three crucial steps enrolled with inflammation after injury, the early pro-inflammatory step, pro-inflammatory response, and a final stage for restoration of tissue homeostasis when the inflammatory cells either exit the site of injury or are eliminated through apoptosis (29). Hence, the persistence of inflammation in EP-SRP/SR may be the key factor for the worsened parameters when compared with SP-SRP/PSS. Also, the anti-resorptive effect of SR, evidenced by the lower number of TRAP-positive cells in EP-SRP/SR at 30 days, may have compromised the equilibrium between bone formation and bone resorption, jeopardizing the turnover of the alveolar bone. Furthermore, this deficiency in bone turnover could also be confirmed by OCN immunolabeling. The expression of OCN was higher in groups treated with SRP when compared with EP-PSS and EP-SR, however, with no significant differences between EP-SRP/PSS and EP-SRP/SR (group with the lowest number of TRAP-positive cells).

While comparing the studies that evaluated the influence of SR in the periodontium, the differences in the doses used and the methods of EP induction must be emphasized, mainly because nylon threads accumulate less bacterial plaque (30). However, despite these variations, our study corroborated with Karakan *et al*. (6) and Souza *et al*. (14) which observed that SR seems to decrease alveolar bone loss during the progression of EP. On the other hand, the absence of difference in the PBF between EP-SRP/PSS and EP-SRP/SR contradicts the potential benefits of SR as an adjunctive to SRP.

Another topic that might be addressed is the occurrence of side effects caused by the systemic the intake of SR. For SR, they can range from mild complication, such as nausea and diarrhea, which will disappear after approximately three months of use (31) to a potential arrhythmogenic effect as a consequence of alterations on the concentration of plasmatic calcium (32). Therefore, any evidence of SR as adjuvant therapy might be interpreted with caution, because the local positive effects of this anti-resorptive drug in periodontitis may not overcome the potential risks to severe systemic side effects.

## Conclusions

Within the limits of this study and based on the progression and treatment of ligature-induced periodontitis, SR reduced the alveolar bone loss in non-treated animals and presented no standout benefits over the conventional forms of treating EP.
